# Prediction-Augmented Shared Decision-Making and Lung Cancer Screening Uptake

**DOI:** 10.1001/jamanetworkopen.2024.19624

**Published:** 2024-07-01

**Authors:** Tanner J. Caverly, Renda S. Wiener, Kyle Kumbier, Julie Lowery, Angela Fagerlin

**Affiliations:** 1 Center for Clinical Management Research, Department of Veterans Affairs Ann Arbor Healthcare System, Ann Arbor, Michigan; 2 Department of Learning Health Sciences, University of Michigan Medical School, Ann Arbor; 3 The Pulmonary Center, Boston University School of Medicine, Boston, Massachusetts; 4 Center for Healthcare Organization and Implementation Research, Edith Nourse Rogers Memorial Veterans Hospital, Bedford, Massachusetts; 5 University of Utah School of Medicine, Salt Lake City; 6 Informatics Decision-Enhancement and Analytic Sciences (IDEAS) Center for Innovation, Department of Veterans Affairs Salt Lake City Healthcare System, Salt Lake City, Utah

## Abstract

**Importance:**

Addressing poor uptake of low-dose computed tomography lung cancer screening (LCS) is critical, especially for those having the most to gain—high-benefit persons with high lung cancer risk and life expectancy more than 10 years.

**Objective:**

To assess the association between LCS uptake and implementing a prediction-augmented shared decision-making (SDM) tool, which enables clinicians to identify persons predicted to be at high benefit and encourage LCS more strongly for these persons.

**Design, Setting, and Participants:**

Quality improvement interrupted time series study at 6 Veterans Affairs sites that used a standard set of clinical reminders to prompt primary care clinicians and screening coordinators to engage in SDM for LCS-eligible persons. Participants were persons without a history of LCS who met LCS eligibility criteria at the time (aged 55-80 years, smoked ≥30 pack-years, and current smoking or quit <15 years ago) and were not documented to be an inappropriate candidate for LCS by a clinician during October 2017 through September 2019. Data were analyzed from September to November 2023.

**Exposure:**

Decision support tool augmented by a prediction model that helps clinicians personalize SDM for LCS, tailoring the strength of screening encouragement according to predicted benefit.

**Main outcome and measure:**

LCS uptake.

**Results:**

In a cohort of 9904 individuals, the median (IQR) age was 64 (57-69) years; 9277 (94%) were male, 1537 (16%) were Black, 8159 (82%) were White, 5153 (52%) were predicted to be at intermediate (preference-sensitive) benefit and 4751 (48%) at high benefit, and 1084 (11%) received screening during the study period. Following implementation of the tool, higher rates of LCS uptake were observed overall along with an increase in benefit-based LCS uptake (higher screening uptake among persons anticipated to be at high benefit compared with those at intermediate benefit; primary analysis). Mean (SD) predicted probability of getting screened for a high-benefit person was 24.8% (15.5%) vs 15.8% (11.8%) for a person at intermediate benefit (mean absolute difference 9.0 percentage points; 95% CI, 1.6%-16.5%).

**Conclusions and Relevance:**

Implementing a robust approach to personalized LCS, which integrates SDM, and a decision support tool augmented by a prediction model, are associated with improved uptake of LCS and may be particularly important for those most likely to benefit. These findings are timely given the ongoing poor rates of LCS uptake.

## Key Points

**Question:**

How is using a prediction-augmented shared decision-making tool that helps clinicians personalize the strength of their lung cancer screening (LCS) recommendations associated with LCS uptake?

**Findings:**

In a 6-site quality improvement study with 9904 participants, LCS uptake following implementation of the tool increased, particularly for high-benefit persons. Mean (SD) predicted probability of receiving LCS for a high-benefit person was 24.8% (15.5%) vs 15.8% (11.8%) for a person at intermediate benefit (mean absolute difference, 9.0 percentage points).

**Meaning:**

These findings suggest personalized SDM tools may be able to improve on the low rate of LCS uptake, especially for those with the most to gain.

## Teaser

This quality improvement study evaluates the association between implementing a prediction-augmented shared decision-making tool and lung cancer screening uptake among US veterans.

## Introduction

Despite national guidelines recommending low-dose computed tomography lung cancer screening (LCS) for those meeting age and smoking criteria since 2013, uptake has remained suboptimal, with less than 20% of eligible persons screened.^1,2,3,4,5,6,7^ Addressing poor LCS uptake is especially important for those having the most to gain: high-benefit persons with high lung cancer risk and high life expectancy (eg, >10-year life expectancy).^8,9,10^ Prediction models can accurately identify such persons^8,11^ and may be an important tool for optimizing the delivery of LCS to those with the most to gain.^12,13^ Indeed, national guidelines from the US Preventive Services Task Force (USPSTF)^10^ and American College of Chest Physicians (CHEST)^9^ have noted modeling studies that demonstrate the potential for prediction-based approaches to improving racial disparities in LCS eligibility and avert more lung cancer deaths compared with using the current, simple risk factor–based eligibility criteria alone (age 50-80, ≥20 pack-year tobacco history, and <15-year quit history).

There remain concerns, however, that implementing prediction into busy primary care settings may not be feasible and could even hinder broad implementation of LCS if completely replacing simple selection criteria.^10^ The 2021 CHEST guideline, on the other hand, has recommended using prediction to augment rather than replace simple criteria, to optimize shared decision-making (SDM) for high-benefit LCS candidates while still allowing for selection of a broad population using simple criteria.^9^

The association between LCS uptake and taking a prediction-augmented approach to SDM for LCS needs further empirical study given limited evaluation of this approach to date. We conducted a multisite interrupted time series study to examine screening uptake following implementation of an SDM tool that alerts clinicians to encourage screening more strongly for patients predicted to be at high benefit.

## Methods

### Overview

This project was part of a Veterans Affairs (VA) Quality Enhancement Research Initiative, was considered nonresearch quality improvement (QI) based on VA policy, and was declared as nonresearch QI activity by the VA Ann Arbor Healthcare System institutional review board.^14^ A waiver of informed consent was granted for this study. Investigators used a fully deidentified dataset for all analyses (ie, they were blinded to patient, clinician, and site). This report follows the Standards for Quality Improvement Reporting Excellence (SQUIRE) reporting guideline for quality improvement studies.

This study was part of a multisite VA QI program to implement prediction-augmented LCS. Prior studies^15,16^ describe the LCS Decision Precision (DP) tool, strategies to increase its adoption, and methods to identify smoking history, LCS conversations, and LCS receipt among those eligible. In a prior study,^15^ we found that primary care clinicians did not use the tool routinely, but screening coordinators used it frequently (tool used a mean [SD] of 246 [164.9] times over 6 months at sites with a screening coordinator vs 3.5 [3.7] times at sites without a screening coordinator; *P* = .049; data collected manually by the coordinators confirming the difference was due to screening coordinator use of the tool). This study presents a planned evaluation of the outcomes of tool use, which was planned a priori in addition to the previously published evaluation of implementation strategies to promote tool use.^15^ A quasi-experiment is an observational evaluation of an intervention (intervention is not allocated randomly) that uses some features of experiments to improve the internal validity of the evaluation. In this case, we used an interrupted time-series study design (accounting for linear time-trends) and comparison of the effect of LCS on uptake for patients estimated by prediction at high benefit vs intermediate benefit, as described in the following section.

### Intervention

**Figure 1.**
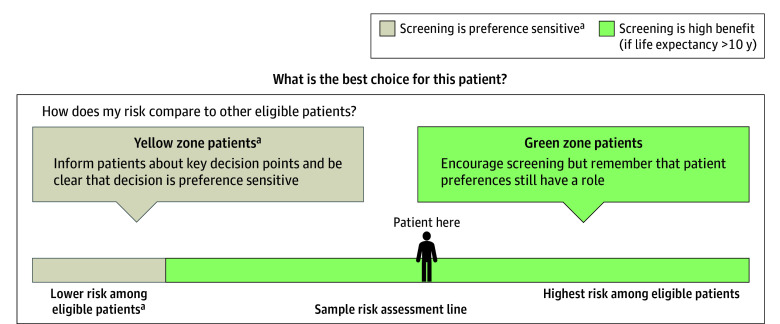
How Clinicians Were Trained to Frame Results for High-Benefit Persons ^a^Best option depends on patient preferences.

Briefly, the tool seeks to help primary care physicians (PCPs) and screening coordinators quickly personalize LCS conversations based on the patient’s combination of risk factors, more strongly encouraging screening for those with higher predicted lung cancer risk and potentially larger health gains with LCS. The tool provided (1) individualized information about screening’s benefit using a well-validated lung cancer risk prediction model^17^; (2) recommendation categories that clarify when screening is and is not high benefit, based on prior published work (Figure 1)^8^; (3) patient-facing graphics and information to aid in the SDM conversation; and (4) support for documenting the SDM discussion (see eFigure 1 in Supplement 1 for screenshots of the tool). During academic detailing visits with PCPs and learning sessions with screening coordinators,^15^ clinicians were given the evidence-based rationale for using a prediction-augmented approach, how to use the tool to personalize SDM for LCS, and how it helps them quickly identify high-benefit candidates for stronger encouragement to screen (see Figure 1 and the full academic detailing pamphlet presented in eFigure 2 in Supplement 1). The detailer emphasized the important role of patient preferences, especially for eligible patients in the preference-sensitive zone, who are predicted to have an intermediate level of benefit with a fine balance between pros and cons, and for whom the best action is highly dependent on how the person weighs the pros and cons of LCS. Screenshots of the study version of the tool are presented in eFigure 1 in Supplement 1. A link to the decision tool was embedded within the clinical reminders for PCPs and screening coordinators at all sites between August 29, 2017, and October 4, 2017 (this date defines tool introduction and the start of the sites’ implementation period). Since completion of this QI project, the tool has been further updated and the current stand-alone web-based version is available online.^18^

### Setting

Eight VA sites were recruited to participate in our QI project. As previously described,^15^ sites were selected based on using a standard set of clinical reminders that prompt primary care teams and screening coordinators to (1) assess a person’s eligibility for LCS based on age and tobacco pack-year and quit history, and (2) carry out SDM discussions to assess for clinical exclusions and whether the patient agrees to enroll into the site’s LCS program. Of the 8 sites recruited into the project, 2 sites were observed to have little to no tool use. Thus, to examine the association between tool use and screening uptake in the current article, we analyzed data from the 6 sites where tool use was observed.

After being introduced to the tool, primary care clinicians and screening coordinators at each site were asked to use the tool within the context of their current screening policies and usual care workflows (standard care). Standard care across all study sites included use of 2013 USPSTF eligibility criteria for LCS: offer screening to patients aged 55 to 80 years with a 30 or more pack-year smoking history and either currently smoking or quit within the past 15 years. We were not able to track in this study who used the tool and whether the tool was used for a specific patient.

### Data Sources

The VA Corporate Data Warehouse (CDW) stores patient information captured through the electronic health record, including information captured in the LCS clinical reminders used at study sites (see eTable 1 in Supplement 1 for CDW variable definitions). LCS clinical reminders capture a patient’s detailed smoking history (smoking status, smoking duration in years, smoking intensity in packs-per-day, and quit history), a clinician’s judgment about the patient’s appropriateness for LCS, and the outcome of the patient-clinician LCS conversation (ie, whether the patient agreed to undergo LCS or decided to defer LCS) (details on clinical reminders in eMethods in Supplement 1).

### Study Sample

The study cohort included patients without a history of low-dose computed tomography LCS who were LCS-eligible (ie, met USPSTF eligibility criteria at the time and not documented to be an inappropriate candidate for LCS by a clinician) during the study period (first fiscal quarter of 2017 through the fourth fiscal quarter of 2019). A patient’s date of study entry was based on approximating when the LCS decision likely occurred: (1) if an LCS conversation was documented close to screening computed tomography, we used the date of the documented conversation; (2) if a person was screened more than 180 days after a documented conversation or was screened in the absence of a documented conversation (319 of 1084 patients screened), the date of the computed tomography examination was assumed the closest approximation; (3) if a person never received screening and no LCS conversation was documented (6178 of 8820 patients never screened), we used the date of the first documented LCS pack-year eligibility assessment.

### Statistical Analysis

A multilevel logistic regression model was fit with receipt of screening as the outcome, adjusting for linear time trend, site, and patient characteristics (age, sex, self-reported race [Black, White, and other (American Indian or Alaska Native, Asian, and Hawaiian or Pacific)], comorbidities, driving distance, and individualized lung cancer risk).^11^ Variables for the model were selected because previous research has shown they are associated with screening uptake and/or are known factors that influence LCS benefit (ie, lung cancer risk factors and comorbidities that impact life expectancy).^16^ A person's annual lung cancer incidence risk was estimated using the Bach et. al. prediction model,11 with age, sex, years of smoking duration, and average packs-per day of smoking (during years smoked) as the model inputs (no asbestos exposure assumed due true exposure being rare and this information not being routinely available). Based on prior work, predicted benefit was categorized in the analysis as it was presented to clinicians in the tool: (1) high benefit (due to high annual lung cancer risk between 0.3%-1.3%); and (2) intermediate benefit (preference-sensitive) due to lower lung cancer risk (<0.3%) or due to high competing mortality.^8^ Methods for calculating individualized lung cancer risk and categorizing predicted benefit are further detailed in the eMethods in Supplement 1.

A variable indicating tool implementation period and period-specific versions of a variable to describe the outcomes of predicted benefit on uptake (benefit-based LCS) were also included. Each site’s 3-month implementation period started on the date the tool was introduced at that site. Benefit-based LCS was a prespecified primary analysis for the study, as described in our prior study evaluating implementation strategies^15^ and on clinicaltrials.gov (benefit-based LCS described as precision decision making, clinicaltrials.gov ID NCT02765412). Benefit-based LCS (the association between a person's predicted benefit and LCS uptake) was estimated for each period. See eMethods in Supplement 1 for further detail on tool implementation period and our benefit-based LCS analysis (primary analysis). We also examined potential low-value screening during the 3 study time periods, defined as occurring when a person (1) did not meet 2018 USPSTF eligibility criteria and (2) is not predicted to be at high benefit by our model. Significance was set at a *P *value less than .05. Analyses for this paper were performed September to November 2023 in R version 4.3.1 (R Project for Statistical Computing) and SAS version 9.4 (SAS Institute)t

##  Results

**Figure 2.**
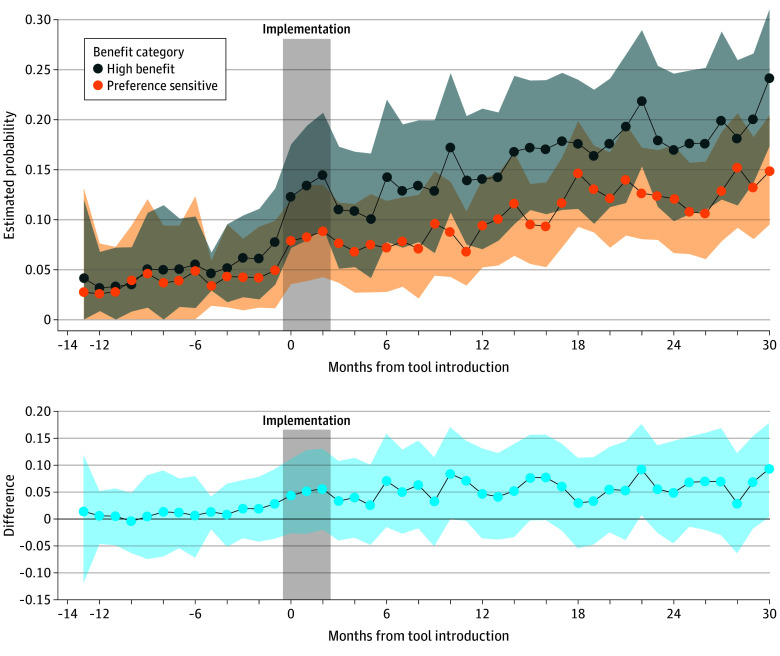
Predicted Probability of Uptake of Low-Dose Computed Tomography Lung Cancer Screening by Month Shaded areas indicate 95% CIs (calculated by running a simulation 100 000 times).

Our study cohort included 9904 patients with a median (IQR) age of 64 (57-69) years. In this group, 9277 (94%) were male, 1537 (16%) were Black, 8159 (82%) were White, and 208 (2%) were other races (see Table 1 for more details on study cohort characteristics). Among this study cohort, 1084 (11%) received screening. Based on their combination of risk factors, 5153 patients (52%) were predicted to be at intermediate (preference sensitive) benefit and 4751 (48%) were at high benefit. Table 2 details the association between covariates and uptake in the cohort. Findings were similar to past results.^16^ The likelihood of being screened increased over time (odds ratio [OR], 1.06; 95% CI, 1.05-1.07; indicating a mean [SD] 6% [0.6%] increase in LCS uptake per month during the study) and the odds of screening were lower in those with an estimated life expectancy of less than 10 years (OR, 0.39; 95% CI, 0.22-0.69). The association between predicted benefit on LCS uptake was greater after tool implementation; that is, more benefit-based LCS. Table 2 and Figure 2 show the rapid increase in benefit-based LCS with early implementation. This association was sustained, and by the final month of the study, the mean (SD) estimated probability of getting screened for a high-benefit person was 24.8% (15.5%) vs 15.8% (11.8%) for a person at intermediate (preference-sensitive) benefit (mean absolute difference, 9.0 percentage points; 95% CI, 1.6%-16.5%).

**Table 1.** Characteristics of the Study Cohort

Characteristic	No. (%) (N = 9904)
Age at study entry, median (IQR), y	64 (57-69)
Sex	
Male	9277 (94)
Female	627 (6)
Race	
White	8159 (82)
Black	1537 (16)
Other^a^	208 (2)
Charlson comorbidities	
0	6512 (66)
1	1502 (15)
≥2	1890 (19)
At a VA Medical Center	2182 (22)
Distance to facility, miles	38 (13-90)
Risk group	
High benefit	4751 (48)
Preference sensitive	5153 (52)

Abbreviation: VA, Department of Veterans Affairs.

^a^ Other includes Asian, Alaska Native or American Indian, and Hawaiian or Pacific Islander.

**Table 2.** Association Between Study Cohort Demographics and the Outcome of Screening Utilization

Covariate	Adjusted OR (95% CI)
Age, y	0.96 (0.94-0.97)
Sex	
Male	1 [Reference]
Female	1.32 (0.96-1.81)
Race (electronic health record)	
White	1 [Reference]
Black	0.80 (0.64-1.01)
Other^a^	0.85 (0.52-1.39)
Charlson Comorbidities, count	0.99 (0.93-1.04)
At a central VA Medical Center	
No (outlying VA clinic)	1 [Reference]
Yes	1.37 (1.07-1.75)
Distance, miles (log-transformed)	0.93 (0.86-0.99)
Limited life expectancy	
No	1 [Reference]
Yes	0.39 (0.22-0.69)
Calendar time, in months since start of QI program	1.06 (1.05-1.07)
Implementation period	
Preimplementation	1 [Reference]
Implementation (3 mos)	41.3 (5.04-338)
Postimplementation	13.1 (3.34-51.5)
Primary analysis: period-specific estimate of the association between predicted benefit and LCS receipt (benefit-based LCS)^c^	
Benefit-based LCS during preimplementation period	1.03 (0.82-1.31)
Benefit-based LCS during implementation period	1.81 (1.34-2.46)
Benefit-based LCS during postimplementation period	1.55 (1.41-1.72)

Abbreviations: LCS, lung cancer screening with low-dose computed tomography; OR, odds ratio; QI, quality improvement; VA, Department of Veterans Affairs.

^a^ Other includes Asian, Alaska Native or American Indian, and Hawaiian or Pacific Islander.

Figure 2 also shows how uptake increased for the intermediate benefit (preference-sensitive) group during the study time period as well, although to a lesser degree than the high-benefit group. Finally, to look for unintended consequences of tool implementation, we examined the rate of potential low-value LCS uptake (screening when ineligible by USPSTF criteria and not high-benefit by prediction) during each study period (see eResults and eTable 2 in Supplement 1). Low-value LCS uptake was low during all time periods in this cohort and appeared to decrease, if anything, after tool implementation: 32% of potential low-value persons screened during the preperiod (6 screened out of 19 found to be potentially low value) and 7% during the postperiod (71 screened out of 1057 potentially low value).

## Discussion

Identifying tools and approaches that may be able to increase LCS uptake, especially for those with the most to gain, is timely. Only 10% to 20% of eligible persons receive LCS.^10,11^ Moreover, recent studies indicate that uptake is poorly distributed: veterans with high lung cancer risk and thus higher predicted benefit from LCS, including Black veterans, are less likely to be screened, and veterans with lower benefit due to poor health and limited life expectancy are more likely to be screened.^1,16^ In another study, clinicians responsible for delivering LCS clearly endorsed the need for help in identifying who could benefit more from LCS, and which patients were poor candidates.^19^ Learning how to feasibly implement tools like DP into routine clinical practice across diverse health systems may be an important way to improve LCS uptake, increase the equitable delivery of LCS, and optimize LCS’s ability to improve lung cancer outcomes.

Our quasi-experimental findings suggest that implementing a robust approach to personalized LCS, which integrates SDM and a decision support tool augmented by a prediction model, is associated with improved screening uptake and may be particularly important for those most likely to benefit. The sustained, higher rate of uptake among high-benefit persons in this study reflects the tool’s emphasis on identifying high-benefit persons for stronger encouragement to get screened. The increased uptake among high-benefit persons did not come at the cost of reducing uptake among eligible persons at intermediate benefit (for whom screening is still appropriate if aligned with the patient’s personal preference). Meanwhile, after tool implementation, there were substantially lower odds of LCS among people with less than 10 years of life expectancy, suggesting the tool’s ability to also reduce low-value screening. These findings are highly aligned with how the decision tool was designed (Figure 1).

The tool’s association with increased LCS uptake is supported by similar findings outside the VA health system. In a previously published study examining the feasibility and impact of using clinician-facing electronic health record (EHR) prompts and an EHR-integrated version of the tool, which automates risk calculations and is better integrated into primary care workflows,^20^ there was a 4.7-fold increase (95% CI, 3.1-7.1) in the odds of completing LCS postintervention compared with the preintervention phase. These studies suggest that this tool may be a viable path to increasing LCS uptake through taking a personalized approach to SDM. Furthermore, the personalized SDM supported by the tool in this study is based on a general framework called everyday SDM,^21^ and was well-received by LCS-eligible adults in a prior study.^22^

Multiple covariates had CIs that did not cross 1: age, being at a central VA Medical Center, distance from VA facility where screening is completed, having a limited life expectancy, calendar time in months since start of QI program, implementation period, and period-specific estimates of the effect of predicted benefit on LCS receipt (benefit-based LCS, primary analysis) during implementation and postimplementation period. As in a prior study,^16^ older age and longer distance to the central VA medical facility was associated with a lower odds of screening uptake, while getting primary care at a central facility offering screening was associated with higher odds of uptake.

### Limitations

This study has limitations. The overall increase in LCS uptake that was sustained throughout the study could well have been influenced by other initiatives at the study sites outside of the tool implementation. Each of these sites had committed resources and infrastructure to systematically rolling out LCS to their eligible patients, including LCS clinical reminders and dedicated screening coordinators. The finding of comparatively higher LCS uptake among high-benefit patients, on the other hand, is a stronger quasi-experimental finding that is difficult to attribute to factors outside of our tool implementation. It is unlikely that clinicians could accurately estimate individualized LCS benefit without using a tool or risk calculator,^19^ and our implementation evaluation did not identify routine use of other tools outside our tool during the study time period.^15^ Another limitation is that these findings are a function of dedicated screening coordinators using the tool, rather than primary care clinicians who are on the frontline for identifying LCS candidates and have the widest patient reach. As mentioned previously, an updated version of the tool that automates calculations and is better integrated into primary care workflows is currently available and being used by multiple health systems.^20^ Further study is needed to examine the feasibility of disseminating tools like this more broadly—and the feasibility of implementing a prediction-augmented approach to SDM across a diverse set of health systems.

## Conclusions

We found that, following implementation of a robust approach to personalized SDM for LCS, people with higher anticipated benefit from LCS had a greater increase in screening uptake than those with intermediated benefit. This quasi-experimental finding supports the use of prediction to optimize the delivery of LCS. A cluster-randomized or stepped-wedge randomized trial testing the feasibility of implementing prediction-augmented LCS across diverse health systems is needed to further assess the scalability of this approach.

## Notes

Supplement 1. **eTable 1. **CDW Variables and Their Definitions **eTable 2. **Potential Low-Value Lung Cancer Screening by Implementation Period **eFigure 1. **Decision Precision (DP) Tool/Intervention Screenshots **eFigure 2. **Academic Detailing Pamphlet
eMethods.

eResults.

eReferences.

Supplement 2.
Data Sharing Statement
