# Supplementary material for: Prediction-Augmented Shared Decision-Making and Lung Cancer Screening Uptake
Source: JAMA Netw Open. 2024 Jul 1;7(7):e2419624. doi: 10.1001/jamanetworkopen.2024.19624 (PMC12312416; doi:10.1001/jamanetworkopen.2024.19624)
Supplement: Supplement 1. — eTable 1. CDW Variables and Their Definitions eTable 2. Potential Low-Value Lung Cancer Screening by Implementation Period eFigure 1. Decision Precision (DP) Tool/Intervention Screenshots eFigure 2. Academic Detailing Pamphlet eMethods. eResults. eReferences. [file jamanetwopen-e2419624-s001.pdf]

Supplemental Online Content

Caverly TJ, Wiener RS, Kumbier K, Lowery J, Fagerlin A. Prediction-augmented shared decision making and lung cancer screening uptake. *JAMA Netw Open*. 2024;7(7):e2419624. doi:10.1001/jamanetworkopen.2024.19624

eTable 1. CDW Variables and Their Definitions

eTable 2. Potential Low-Value Lung Cancer Screening by Implementation Period

eFigure 1. Decision Precision (DP) Tool/Intervention Screenshots

eFigure 2. Academic Detailing Pamphlet

eMethods.

eResults.

eReferences.

This supplemental material has been provided by the authors to give readers additional information about their work.

© 2024 Caverly TJ et al. *JAMA Network Open.*

eTable 1. CDW Variables and Their Definitions

Variable Definitions in the VA's Corporate Data Warehouse (CDW)

Variable	CDW domain	Code/Definition
Years smoked	Health Factors	Health Factor type=LCS YEARS SMOKED
Packs Smoked	Health Factors	Health Factor type=LCS PACKS/DAY
Quit Time	Health Factors	Health Factor type=LCS YEAR QUIT SMOKING/LCS QUIT YEAR (ACTUAL)/LCS QUIT DATE
Smoking Status	Health Factors	Health Factor type=LCS CURRENT SMOKER/LCS FORMER SMOKER
LCS Exclusions	Health Factors	Health Factor type=LCS HAS EXCLUSIONS/LCS NO EXCLUSIONS
LCS decision	Health Factors	Health Factor type=LCS AGREES TO BE SCREENED
LCS Reminder Location	Health Factors/Outpatient Visit	Sta6a facility code
Age, gender, race	Patient	*Included only self-identified race records
Charlson Comorbidity index	Inpatient & Outpatient Diagnosis	Published codes
Zip Code; Latitude; Longitude	Patient Address	N/A
Distance to central facility[a]	Calculated using SAS GeoDist function	N/A
CT Screen	Outpatient	CPT Code 71250

[a] Distance to the central medical facility was calculated using the latitude and longitude of patients' home address and the latitude and longitude of the VA medical facilities offering LCS (shortest geographic distance, not driving distance).

© 2024 Caverly TJ et al. *JAMA Network Open.*

eTable 2. Potential Low-Value Lung Cancer Screening by Implementation Period

	Number of people receiving potentially low-value lung cancer screening		
	Pre-implementation	Implementation	Post-implementation
Screened	6	1	71
Not screened	13	94	986

© 2024 Caverly TJ et al. *JAMA Network Open.*

eFigure 1. Decision Precision (DP) Tool/Intervention Screenshots

DP Home screen

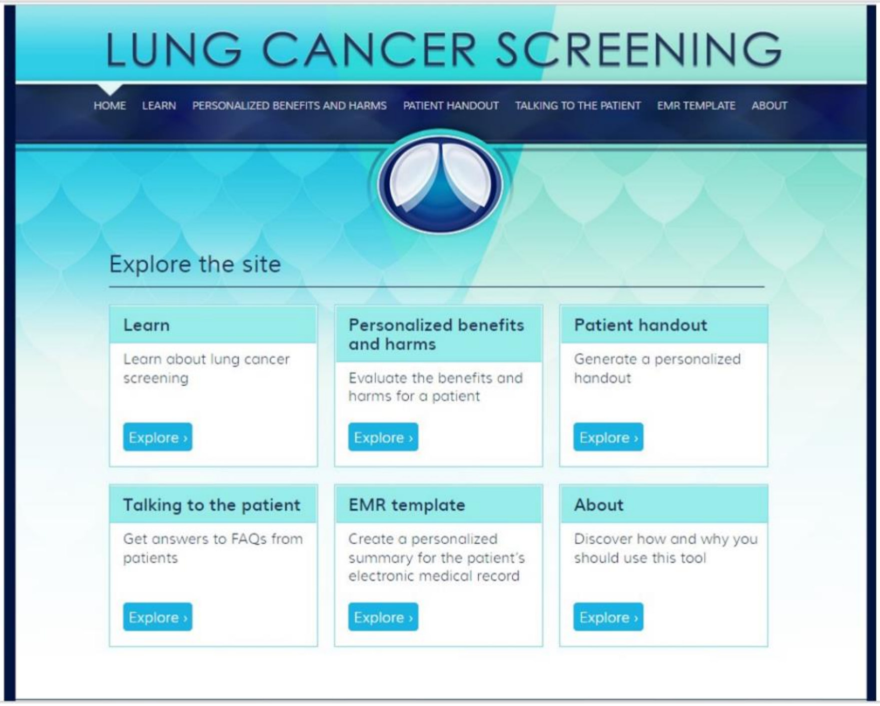

Risk Factor Entry Screen

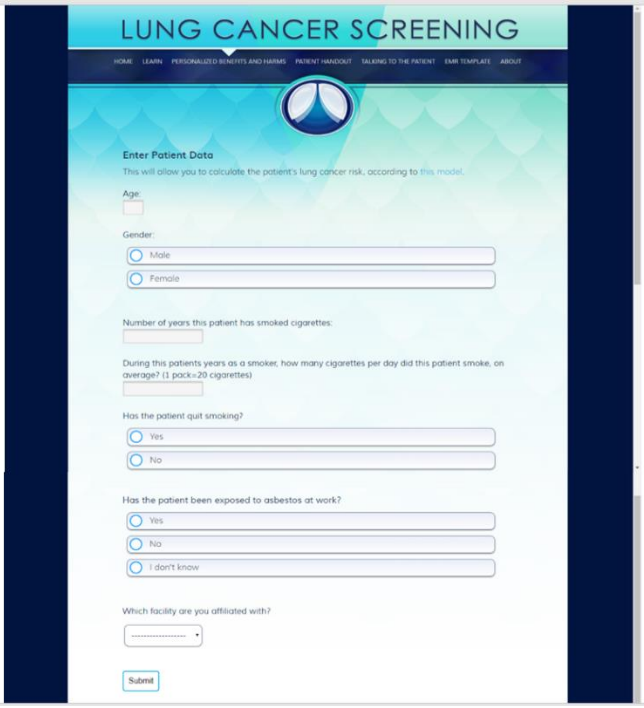

© 2024 Caverly TJ et al. *JAMA Network Open.*

High Benefit Patient Output

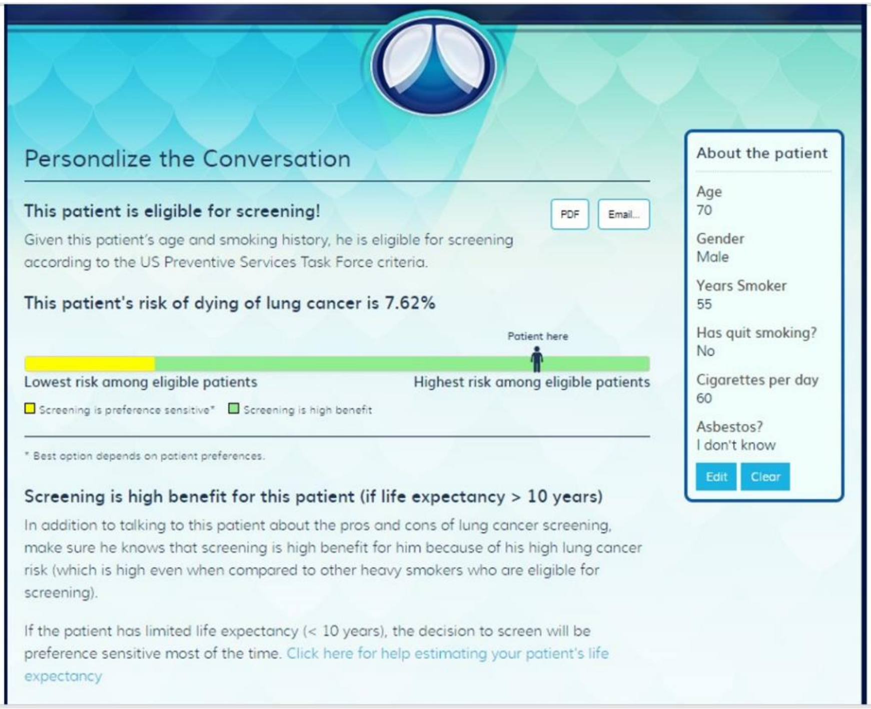

High Benefit Patient Output (cont.)

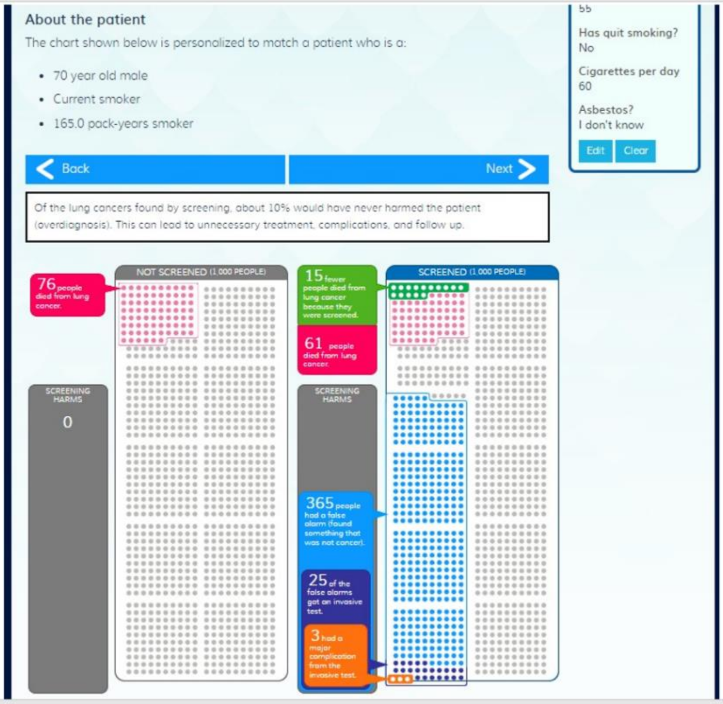

© 2024 Caverly TJ et al. *JAMA Network Open.*

Preference Sensitive Patient EHR Template

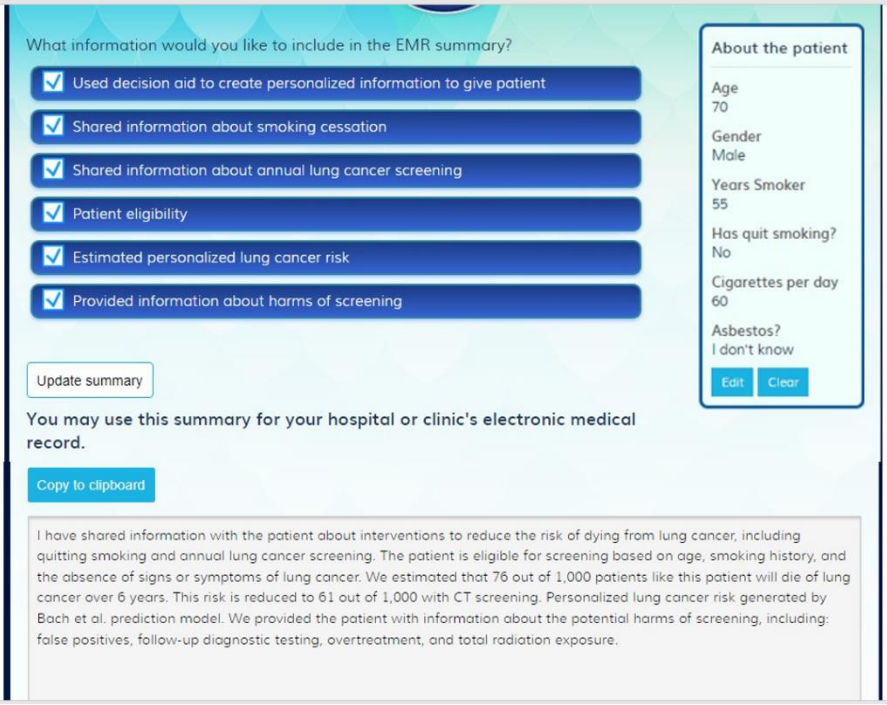

Preference Sensitive Patient Output

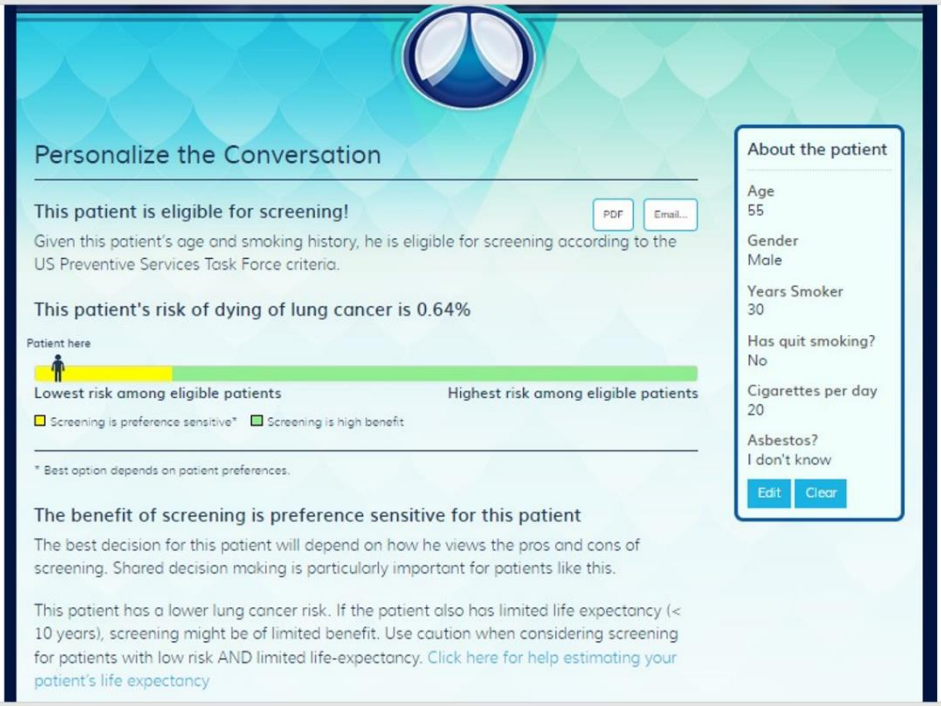

© 2024 Caverly TJ et al. *JAMA Network Open.*

Preference Sensitive Patient Output (cont.)

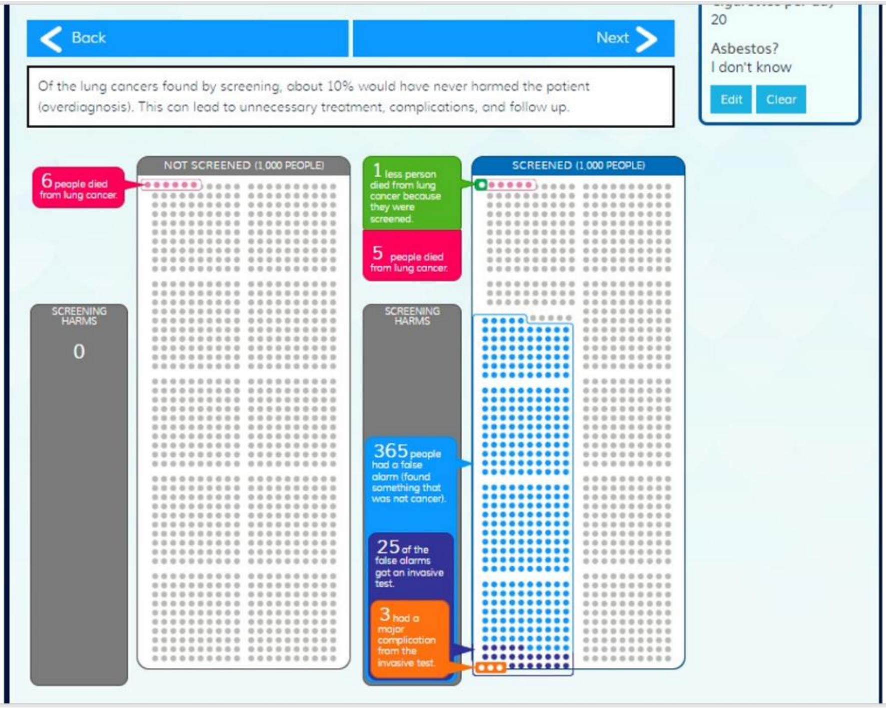

How to talk to your patient

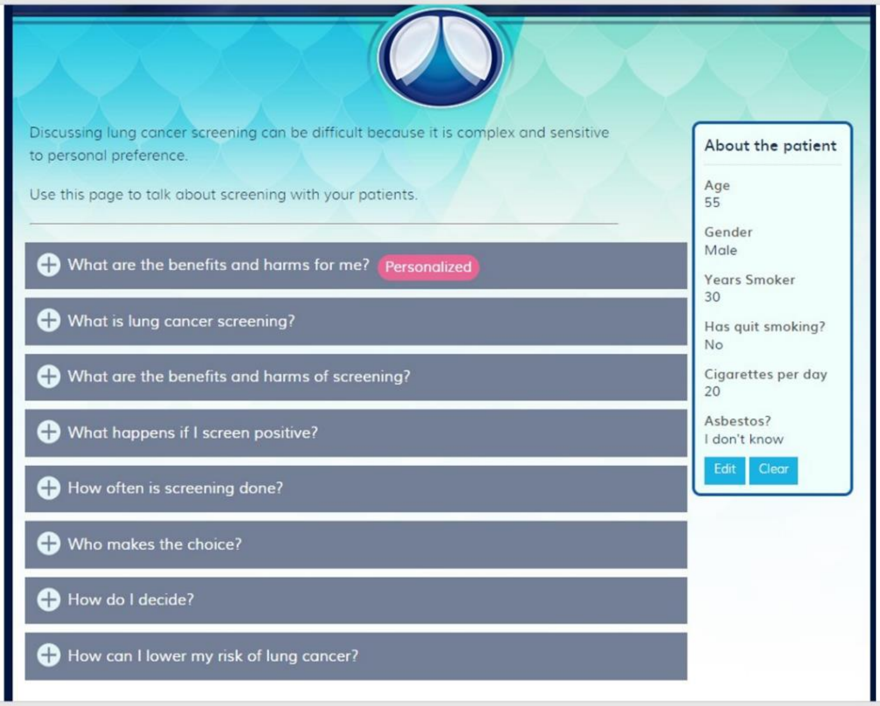

© 2024 Caverly TJ et al. *JAMA Network Open.*

How to talk to your patient: What are the benefits and harms of screening?

What are the benefits and harms of screening?

Benefits and harms

Benefits:

Based on research, if a group of heavy smokers is screened once a year, fewer people would die of lung cancer after 6 years. This is because screening can help find lung cancer in an earlier, more treatable stage.

Harms:

- **False alarms**: Lung cancer screening may find something in your lungs turns out *not* to be lung cancer ("false positives"). Patients can feel stressed or anxious while awaiting the results of further testing.
- **Invasive Procedures**: Sometimes false alarms lead to extra testing that involves putting a tube in the body or having surgery ("invasive procedures").
- **Complications** of extra testing: Invasive procedures can sometimes cause harm to the patient ("complications"). Complications can include:
  - Bleeding
  - Infection
  - Collapsed lung (rare)
- **Radiation**: The low-dose radiation from lung cancer screening increases your risk of developing cancer years later by a small amount.
- **Overtreatment**: Sometimes screening tests find cancers that would have never caused problems. It is often impossible to tell which cancers will and won't cause problems. With screening, there is a chance someone may be treated unnecessarily with surgery and radiation for a cancer that would not have harmed them.

How to talk to your patient: What is lung cancer screening?

What is lung cancer screening?

About lung cancer screening

- Screening looks for a disease before you have symptoms. It can help find lung cancer in an earlier, more treatable stage.
- A doctor in a radiology department uses a low-dose chest Computerized Tomography scan (CT scan). This CT gives a detailed picture of your lungs.
- You lie on a table with your arms above your head. The table slides into a scanner that's shaped like a big, open doughnut. You will hold your breath for a few seconds during the scan.

How to talk to your patient: What happens if I screen positive?

What happens if I screen positive?

Screening positive

Even if we do find a spot on your lungs, there's a good chance that you do **not** have cancer. 96 out of 100, or 96% of positives are actually false alarms.

- If we do find a spot, you will most likely get another CT scan (also called computerized tomography scan), usually between 3 and 12 months later.
- If the follow up scan shows cause for worry, you might get a biopsy. In a biopsy, doctors take a sample of tissue from your body to check for cancer.

Keep in mind: screening is a process, not a one time event.

© 2024 Caverly TJ et al. *JAMA Network Open.*

How to talk to your patient:
How do I decide?

How do I decide?

Deciding whether or not to screen

Let's talk about your feelings about the balance of benefits and harms that can go along with lung cancer screening

Attitude toward your health

On a scale from "watch and wait" to "take care of it", where do you fall?

Watch and wait — Take care of it

- I prefer to do as little as possible when it comes to medicine.
- Given the option, I prefer not to take drugs or get tests.
- Though not necessarily fearful of doctors or medicine I believe that health interventions can have unintended consequences.
- Most of the time the body works just fine on its own, without intervention.
- I prefer to "watch and wait" and often go by the saying "If it ain't broke, don't fix it."

- I prefer to aggressively pursue treatments of various kinds.
- I like doing things that may positively affect my health, such as taking prescription medicines, gettings tests or medical procedures that might help, or taking over-the-counter drugs.
- If there is a way that could fix or address a physical problem, I generally like to do it.
- If there is a way to address a current or future health problem, I will do it and not "watch and wait."

Worry about lung cancer

- How **worried** are you about getting lung cancer?
- How worried would you be about getting lung cancer if you **didn't get screened**?
- Do you think you would **worry less** if you got screened? How much less?

Worry about false alarms

Lung cancer screening can result in false alarms about a third of the time. Some patients experience anxiety about false alarms while awaiting the results of further testing.

How to talk to your patient:
How often is screening done?

How often is screening done?

Screening frequency

- Screening is done yearly, and can reduce your chance of dying from lung cancer.
- Screening less often has not been studied and may not be beneficial.
- Clinicians and patients should regularly re-assess if screening is a good option every 3–5 years.
- Whether you decide to screen today or not, you can decide at any time whether to screen for lung cancer.

How to talk to your patient:
Who makes the choice?

Who makes the choice?

You make the choice

I'm here to help you make the choice, but it's your decision.

How to talk to your patient:
How can I lower my risk of lung cancer?

How can I lower my risk of lung cancer?

Lowering your risk

Regardless of your decision about screening, quitting smoking is the most important thing you can do to lower your chance of dying from a variety of diseases, not just lung cancer. Quitting smoking helps with emphysema and heart and vascular diseases as well.

© 2024 Caverly TJ et al. *JAMA Network Open.*

eFigure 2. Academic Detailing Pamphlet

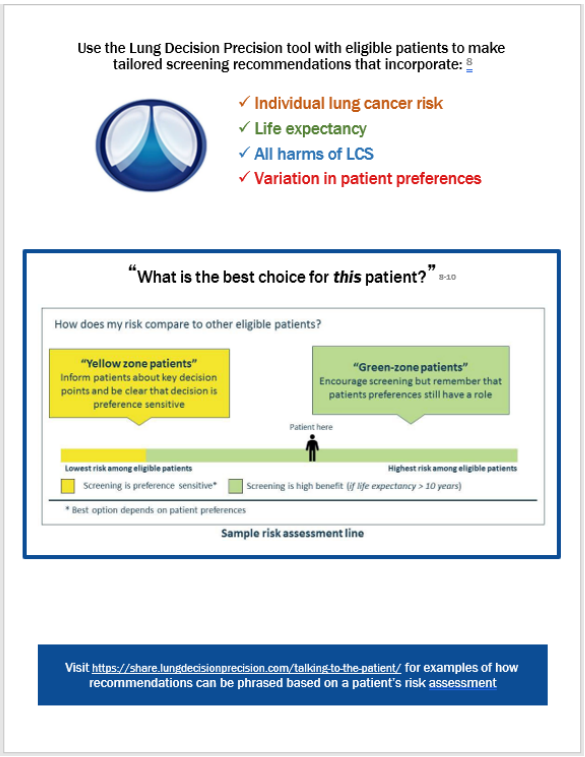

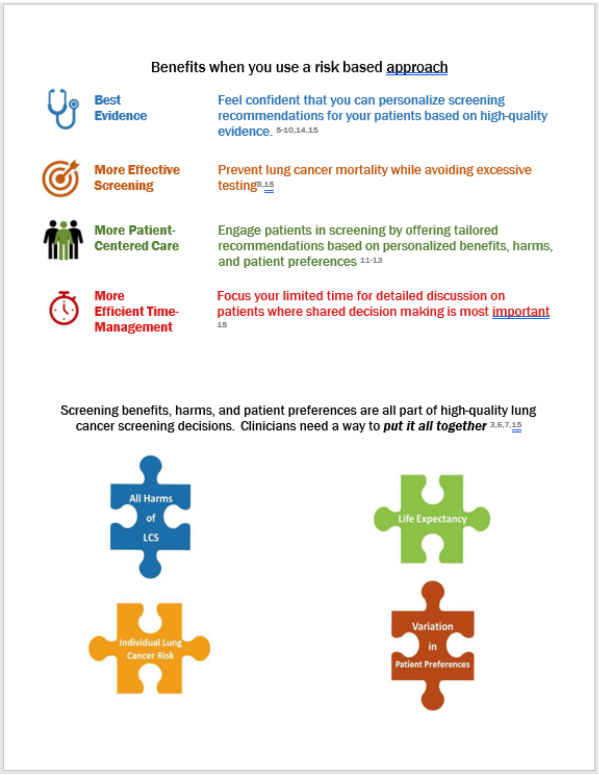

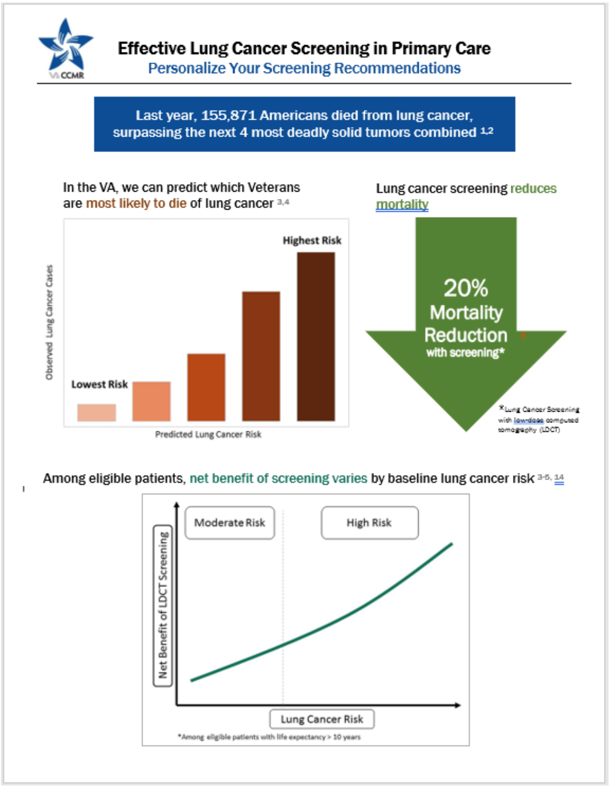

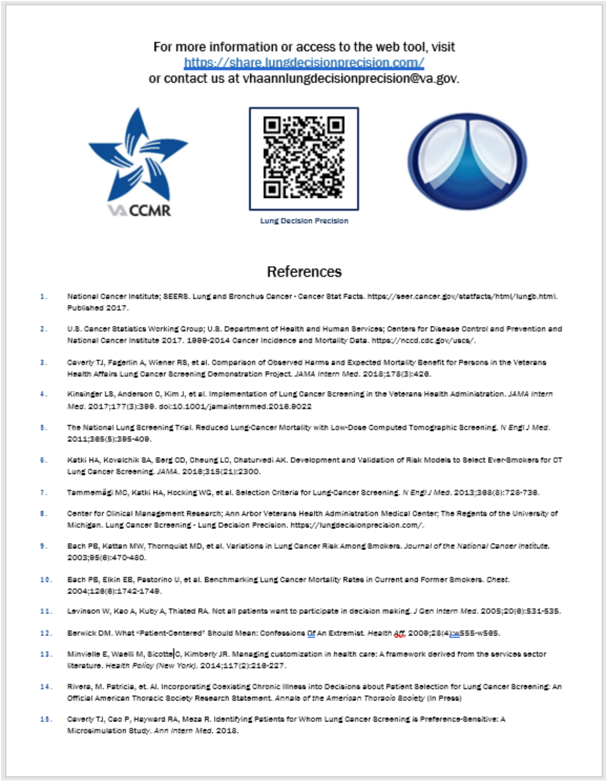

© 2024 Caverly TJ et al. *JAMA Network Open.*

eMethods.

Clinical Reminders for lung cancer screening at the VA
The following is excerpted from a prior report detailing the LCS clinical reminders that capture a patient's smoking history (pack-year reminder) and if & when a documented LCS conversation took place (initial LCS provider reminder):[1]

> These clinical reminders capture patient information through health factors which are stored in the VA Corporate Data Warehouse. Health factor data from 2 of these reminders was utilized in the analysis, the Tobacco Pack-Year Reminder and the Initial Lung Cancer Screen (Provider) Reminder. The Tobacco Pack-Year Reminder was only active for patients who were potentially eligible based on age (55–80 years old), a lack of permanent exclusions (e.g., history of lung cancer, health factor indicated life expectancy <6 months), and had not received a chest CT in the previous year. This reminder captured smoking status, how long patients smoked, and how many cigarettes per day patients smoked on average. These data points were used to calculate a patient's pack-year smoking history which is used to determine "potential LCS eligibility." Pack- year history is the number of average packs of cigarettes smoked per day times the number of years smoked (e.g., average of 1 pack of cigarettes smoked per day x 40 years smoked=40 pack-years). The Tobacco Pack-Year Reminder is completed by primary care staff in most cases. The Initial Lung Cancer Screen (Provider) Reminder is completed primarily by primary care clinicians (PCPs) (although it is possible for others such as screening coordinators to complete it and this did occur) and was active for "potentially LCS eligible" patients based on the completed Tobacco Pack-Year Reminder (i.e., [based on USPSTF criteria at the time of the study] patient is a current smoker or former smoker who quit <15 years ago, and has ≥30 pack-year smoking history). This reminder allowed PCP's to assess a patient's appropriateness for LCS and indicate if patients had any exclusions that would make them a poor LCS candidate (e.g., severe comorbidity; not willing or able to undergo curative lung cancer resection). For patients without exclusions, the reminder also captured a patient's decision to agree to LCS, defer screening for 1 year, or defer screening indefinitely.

Estimating individualized net benefit
Based on prior work,[2] we estimated individualized net benefit using the Bach et. al. risk model, an accurate and well-calibrated model on multiple external validations (inputs: age, sex, asbestos exposure history, and smoking history).[3,4] A patient's model-predicted risk was calculated based on the pack-year record closest to date they appear in our dataset, and then further categorized based on findings from a prior comprehensive microsimulation analysis as high benefit (risk between 0.3% and 1.3%) or intermediate benefit (preference sensitive: i.e., a patient's preferences should alter the recommendation to screen).[2,5] Following this prior work and as discussed in the most recent *CHEST* LCS guideline,[5] the LCS decision was categorized as preference-sensitive for two different reasons: 1) if the person's annual lung cancer risk was predicted to be below 0.3% by the Bach et. al. model, due to being lower-risk/lower-benefit; or 2) if the person's life-expectancy was estimated to be <10 years, because the benefit of screening can be attenuated if the patient also has a limited life-expectancy (even if very high lung cancer risk, due to high competing mortality). Our previously published results have shown that <10-year life-expectancy among heavy-smoking adults can also be predicted using the Bach et. al. model.[2]

Tool Implementation Period and Date of Participant Entry into the Study
The implementation period for the tool lasted 3 months after it was first introduced. The following dates were used to define the beginning of the post-implementation periods at each site: site 1 (6/1/2017), site 2 (6/1/2017), site 3 (2/1/2018), site 4 (1/1/2018), site 5 (1/1/2018), and site 6 (11/1/2017). For assigning date of patient entry into the time-series, our default approach was to have patients appear based on the date of the documented LCS conversation, but there were exceptions in many cases. In the case that a patient was screened more than 180 days after the documented SDM discussion (this occurred about 29% of the time, 320 of the 1091 screened; the median time between the date of the conversation and screening was 51 days, IQR= [26 days, 351 days], max= 1030 days), we decided to use the date of screening receipt, assuming that the actual decision to be screened was likely made closer to the screening date in that case. In the case that a patient never received screening and did not have an LCS conversation documented (which happened about 70% of the time, 6193 of the 8837 never screened), we used the date of the first pack year reminder completed for that patient.

Benefit-Based Screening Analysis

As described in the main text, we utilized an interrupted time series analysis to investigate whether introduction of the tool influenced benefit-based screening. We fit a multilevel logistic regression model where receipt of screening was the outcome of interest. The covariate that makes this an interrupted time series analysis is an indicator variable for the implementation period in which a patient's LCS conversation fell. The beginning of the implementation period and post-implementation period varied by site. A statistically significant coefficient for the period indicator variable would indicate an immediate change in the odds of screening as a result of introducing the tool. The model adjusts for a linear time trend and multiple characteristics including age, gender, race, comorbidities, distance from the site, whether the site was a VA medical center, and, importantly, a patient's individualized estimate of anticipated net benefit with LCS.

We were primarily interested in the interaction between implementation period and individualized benefit. This interaction describes benefit-based LCS, our primary analysis. The interaction resulted in three coefficients in the model, one for each of the three periods. A significant interaction would indicate that the effect of predicted benefit on screening uptake was different from zero during that specific period. This allows interpretations such as the following: “The effect of a person’s predicted benefit on LCS uptake was X during the pre-implementation period; Y during the implementation period; and Z during the post-implementation period.”

© 2024 Caverly TJ et al. *JAMA Network Open.*

eResults.

Study Cohort

Across our 6 study sites, 9,904 people had completed tobacco pack-year information. Of the 9,904 people in the final cohort, 1,084 received screening.

Potential low-value LCS

As described in the main text, potential low value screening was defined as occurring when a person 1) did not meet 2018 USPSTF eligibility crtieria and 2) is not predicted to be at high benefit by our model. Among the 1,084 receiving LCS, 553 (51%) were only predicted to be at only intermediate benefit (not high benefit). A smaller number of the 1,084 receiving LCS were ineligible by USPSTF criteria: 70 were ineligible due to not meeting the 30+ pack-year history; 2 had quit smoking > 15 years ago; and 28 were out of the 55-80 age range (USPSTF criteria at the time of the study).

© 2024 Caverly TJ et al. *JAMA Network Open.*

eReferences.

1. Leishman NJ. Variation in Eligible Patients' Agreeing to and Receiving Lung Cancer Screening: A Cohort Study. *Am J Prev Med*. Published online 2021:9.

2. Caverly TJ, Cao P, Hayward RA, Meza R. Identifying Patients for Whom Lung Cancer Screening is Preference-Sensitive: A Microsimulation Study. *Ann Intern Med*. 2018;169(1):1-9. doi:10.7326/M17-2561

3. Bach PB, Kattan MW, Thornquist MD, et al. Variations in lung cancer risk among smokers. *J Natl Cancer Inst*. 2003;95(6):470-478.

4. Katki HA, Kovalchik SA, Petito LC, et al. Implications of Nine Risk Prediction Models for Selecting Ever-Smokers for Computed Tomography Lung Cancer Screening. *Ann Intern Med*. 2018;169(1):10. doi:10.7326/M17-2701

5. Mazzone PJ, Silvestri GA, Souter LH, et al. Screening for Lung Cancer. *Chest*. 2021;160(5):e427-e494. doi:10.1016/j.chest.2021.06.063

© 2024 Caverly TJ et al. *JAMA Network Open.*
