# Supplementary material for: Prediction-Augmented Shared Decision-Making and Lung Cancer Screening Uptake
Source: JAMA Netw Open. 2024 Jul 1;7(7):e2419624. doi: 10.1001/jamanetworkopen.2024.19624 (PMC12312416; doi:10.1001/jamanetworkopen.2024.19624)
Supplement: Supplement 2. — Data Sharing Statement [file jamanetwopen-e2419624-s002.pdf]

Data Sharing Statement

Caverly. Prediction-Augmented Shared Decision-Making and Lung Cancer Screening Uptake. *JAMA Netw Open*. Published July 01, 2024. doi:10.1001/jamanetworkopen.2024.19624

Data
Data available: No

Additional Information
Explanation for why data not available: VA data restrictions.
